# Androgenic and Estrogenic Response of Green Mussel Extracts from Singapore’s Coastal Environment Using a Human Cell-Based Bioassay

**DOI:** 10.1289/ehp.6990

**Published:** 2004-07-15

**Authors:** Stéphane Bayen, Yinhan Gong, Hong Soon Chin, Hian Kee Lee, Yong Eu Leong, Jeffrey Philip Obbard

**Affiliations:** ^1^Department of Chemistry,; ^2^Tropical Marine Science Institute, and; ^3^Department of Obstetrics and Gynecology, National University of Singapore, Republic of Singapore

**Keywords:** androgen, endocrine disruption, estrogen, green mussel, heavy metals, persistent organic pollutants, reporter gene bioassay, Singapore

## Abstract

In the last decade, evidence of endocrine disruption in biota exposed to environmental pollutants has raised serious concern. Human cell-based bioassays have been developed to evaluate induced androgenic and estrogenic activities of chemical compounds. However, bioassays have been sparsely applied to environmental samples. In this study we present data on sex hormone activities in the green mussel, *Perna viridis*, in Singapore’s coastal waters. *P. viridis* is a common bioindicator of marine contamination, and this study is a follow-up to an earlier investigation that reported the presence of sex hormone activities in seawater samples from Singapore’s coastal environment. Specimens were collected from eight locations around the Singapore coastline and analyzed for persistent organic pollutants (POPs) and heavy metals. Tissue extracts were then screened for activities on androgen receptors (ARs) and estrogen receptors (ER-α and ER-β) using a reporter gene bio-assay based on a HeLa human cell line. Mussel extracts alone did not exhibit AR activity, but in the presence of the reference androgenic hormone dihydrotestosterone (DHT), activities were up to 340% higher than those observed for DHT alone. Peak activities were observed in locations adjacent to industrial and shipping activities. Estrogenic activities of the mussel extract both alone and in the presence of reference hormone were positive. Correlations were statistically investigated between sex hormone activities, levels of pollutants in the mussel tissues, and various biological parameters (specimen size, sex ratio, lipid and moisture content). Significant correlations exist between AR activities, in the presence of DHT, and total concentration of POPs (*r* = 0.725, *p* < 0.05).

Endocrine disruption is now evident at the global scale for humans (Norgil Damgaard et al. 2002), mammals (Kirk et al. 2003), and aquatic organisms (Oberdörster and Cheek 2001). Both natural and anthropogenic chemicals have been implicated as the cause for this problem. Concern is mounting about the number of potential endocrine-disrupting compounds (EDCs) now at large in the biosphere. For instance, 80,000 chemicals are estimated to be in use in U.S. commerce alone, but only a small fraction has been screened for endocrine-disrupting potential (Tully et al. 2000). Estrogens were recently quantified in coastal waters of the United States, and peak concentrations were observed near sources of sewage, highlighting the importance of anthropogenic sources of EDCs in the marine environment (Atkinson et al. 2003). In 2001, the Stockholm Convention under the auspices of the United Nations Environmental Program (UNEP) specified a suite of persistent organic pollutants (POPs) considered as potential EDCs in the environment (UNEP 2001). The “red list” defined at the Stockholm Convention includes dichlorodiphenyltrichloroethane (DDT), chlordanes, lindane, hexachlorobenzene, aldrin, endrin, dieldrin, heptachlor, toxaphene, mirex, and polychlorinated biphenyls (PCBs), dioxins, and furans.

A number of techniques exist for assessing the endocrine effect of anthropogenic chemicals on wildlife. Common assays include *in vivo* tests such as uterine growth bioassays or the use of *in vitro* biomarkers such as vitellogenin proteins, gene transcription, and cell proliferation (Jimènez 1997). So far, no assay has been proven to deliver a comprehensive evaluation of endocrine disruption effects in environmental samples. Furthermore, in most cases, results between assays are not comparable (Jimènez 1997).

Human cell-based gene receptor bioassays enable the comparison of hormonal activity in a sample relative to standard hormones. This type of assay has been principally used to test activities of single congeners such as PCBs (Schrader and Cooke 2003), as well as pesticides (Tully et al. 2000). Reporter gene assays have also been applied to environmental samples such as fresh water (Shen et al. 2001). More recently, Legler et al. (2003) reported the use of a gene receptor bioassay for determining estrogenic activity in sediments and marine organism extracts, including fish and mussels.

In a previous study (Gong et al. 2003), we developed a robust methodology to measure both androgenic and estrogenic activities in seawater samples using a HeLa human cell-based assay. Analysis of samples using this assay revealed that Singapore’s coastal waters displayed high levels of both androgenic and estrogenic activity. This finding poses questions as to the potential biological impact of EDCs in Singapore’s coastal environment. Mussels represent the most common species of shellfish cultivated in the world, with more than 1.1 million tons produced in 1998 (Gosling 1992). The green mussel, *Perna viridis*, is the mussel species naturally prevalent in Asia-Pacific coastal waters (Gosling 1992). As a filter-feeding organism, green mussels have been used as a bioindicator species for various POPs, including organochlorine pesticides (OCPs), PCBs, and polybrominated diphenyl ethers (PBDEs) (Bayen et al. 2003). In this study we report the use of a human cell-based bioassay for the determination of sex hormone activity in extracts of *P. viridis* sampled from Singapore’s coastal waters. Specifically, mussel extracts were screened for hormonal activities on androgen receptors (ARs) and estrogen receptors (ER-α and ER-β), either alone or in the presence of well-known hormones, androgenic dihydrotestosterone (DHT) or estrogenic 17β-estradiol (E_2_). To our knowledge, this study represents the first measurement of both androgenic and estrogenic activities of an environmental biological tissue extract using a human cell-based bioassay. Data on sex hormone activities in *P. viridis* samples collected from the coastal waters of Singapore were then correlated statistically to various parameters measured in the mussels, including contaminant burden, to evaluate the possibility of using this bio-assay as an indicator of the presence of EDCs in biological samples.

## Materials and Methods

### Chemicals.

All organic solvents used for the bioassay were of HPLC grade and were obtained from Fisher Scientific (Fairlawn, NJ, USA) and J.T. Baker (Philipsburg, NJ, USA). We obtained ultrapure water using Nanopure treatment (Barnstead, Dubuque, IA, USA). DHT and E_2_ were purchased from Sigma (St. Louis, MO, USA). Chemicals used for POP and heavy metal analysis have been previously described (Bayen et al. 2003, 2004).

### Green mussel collection and preparation of tissue homogenates.

**Figure 1:**
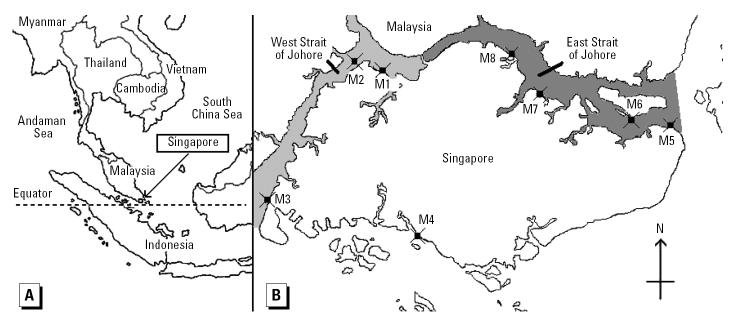
Geographical location of Singapore (*A*) and sampling locations of *P. viridis* (M1–M8) in Singapore’s coastal environment (*B*).

*Perna viridis* specimens were collected from eight sample stations along the coastline of Singapore’s main island between March and April 2002 (Figure 1). Specimens were taken from floating structures and shore defense walls. We collected 20 mussels from each location, but some of these were later rejected so that only the largest specimens and those most similar in size were analyzed for each location. Samples were transported in polyethylene bags in ice boxes to the laboratory for analysis.

In the laboratory, we recorded the sex and size of each specimen. Sex is easily ascertained for *P. viridis* because female tissues are red in color and male tissues are creamy white (Gosling 1992). The soft tissues in the mussel samples were removed from the shell and homogenized in a stainless steel blender to form a single batch sample for each sampling site. These samples were then frozen at −20°C in glass containers.

### Green mussel tissue extraction and human cell-based bioassay.

Green mussel homogenate samples (5.2 ± 0.2 g) were extracted via microwave-assisted extraction using a Mars X oven (CEM, Matthews, NC, USA), with 30 mL methanol/ethanol/dichloromethane/*n*-hexane/ethyl acetate mixture (1:1:1:1:1 by volume). The extraction temperature was increased to 110°C within 10 min and then held for 3 min at this value, using 60% of 1,200 W power. The maximum pressure allowed was set to 200 psi. The extract was then filtered, dried under purified nitrogen, and reresolved in 6 mL methanol/DMSO (1:1 vol/vol). Then 1.2 μL extract was added in 0.6 mL culture media for screening androgenic and estrogenic activities. The cell-based gene receptor bioassay procedure has been described and validated in a previous study (Gong et al. 2003). Briefly, HeLa cells were transiently cotransfected with two plasmids using a lipofectamine technique. The first plasmid consisted of DNA encoding for AR or ER (ER-α and ER-β), and the second an appropriate luciferase reporter gene to drive the androgen response or estrogen response element. After 36 hr incubation, the cells were lysed and collected for measurement of luciferase activity. Bioactivity of the extracts was expressed as percentages of luciferase activity of positive control cells. The gene reporter tests were conducted in duplicate for each sample.

### POP and heavy metal analysis.

The analytical methods for determination of POP and heavy metal concentrations have been reported in previous studies (Bayen et al. 2003, 2004). Briefly, POPs were extracted using accelerated Soxhlet extraction followed by a two-step cleanup procedure that included adsorption chromatography on acid silica gel followed by gel permeation chromatography. Quantification of POPs was performed by gas chromatography/mass spectrometry for 41 PCB congeners, 21 PBDE congeners, *p*,*p*′-DDT, *p*,*p*′-DDD, *o*,*p*′-DDE and *p*,*p*′-DDE, α- and γ-chlordane, mirex, hexachlorobenzene, penta-chloronitrobenzene, and heptachlor.

Samples were digested for heavy metal analysis using an oxidizing acid mixture exposed to microwave energy. Digested solutions were analyzed by inductively coupled plasma/mass spectrometry. Quantification was performed for arsenic, chromium, copper, nickel, lead, zinc, and cadmium.

Validation and quality assurance of the analytical procedure were conducted as described previously (Bayen et al. 2003, 2004). Analytical quality assurance for POPs included a recovery test using ^13^C-labeled PCBs, analysis of standard reference material (SRM2978; National Institute of Standards and Technology, Gaithersburg, MD, USA), reproducibility tests, and standard solution and procedural blank analysis. Quality assurance for heavy metals included recovery tests, reproducibility checks, and procedural blank analysis. Analytical methodology and results were acceptable for the various quality criteria set for individual contaminant determination in the green mussel tissues.

### Data analysis.

All statistical data analyses were performed using XSTAT 6.19 software (Addinsoft, Brooklyn, NY, USA). We assessed differences in the activities for the various sites using the Kruskal-Wallis test, setting an adjusted *p*-value of < 0.05 for significance. Pearson correlation analysis was used to detect any proportional relationships between sex hormone activity and the concentrations of contaminants in the mussel sample tissue. The Pearson correlation *r* coefficient measures the proportional (i.e., linear) relationship between two parameters, where the *r* coefficient varies in the interval [−1.00, +1.00] and a value of 0.00 represents a lack of correlation. Values of −1.00 and +1.00 represent, respectively, perfect negative and positive correlations, respectively. A Pearson matrix of correlation is the summary of all the Pearson coefficients for a specified set of parameters. The significance of the correlation was evaluated for a *t*-test using a *p*-value of 0.05.

## Results

### Endocrine activities of the mussel samples.

**Figure 2:**
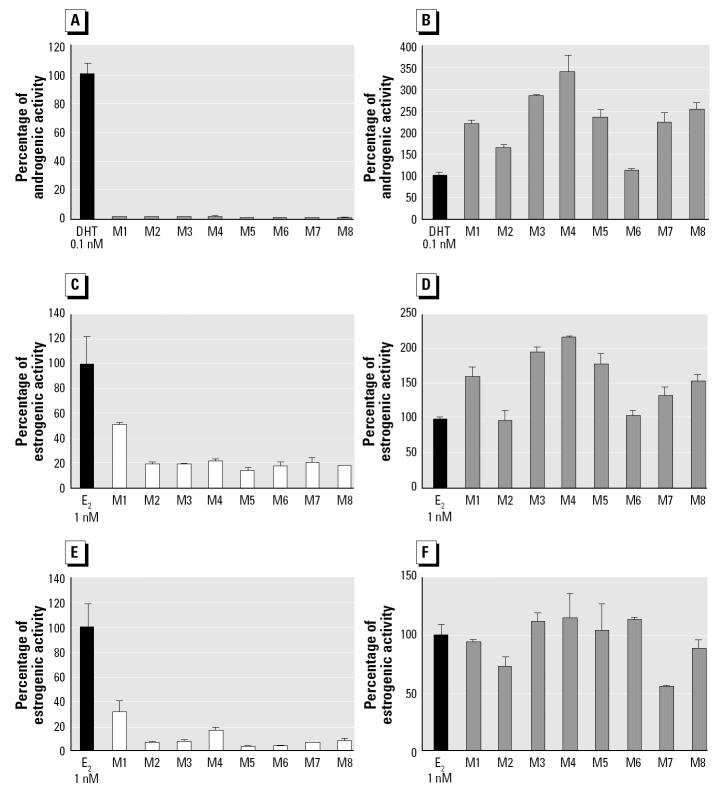
Sex hormone activities of extracts of *P. viridis* (mean ± SD) as a percentage of the reference hormone: AR agonist (*A*) and antagonist (*B*); ER-αagonist (*C*) and antagonist (*D*); and ER-βagonist (*E*) and antagonist (*F*). (*A*), (*C*), and (*E*) represent the activities of the mussel extracts alone. (*B*), (*D*), and (*F*) represent the mussel extracts in the presence of the reference hormone.

Sex hormone activities of *P. viridis* extracts are presented in Figure 2. AR activities in the *P. viridis* extract alone were comparably low between sample locations (< 1% of 0.1 nM DHT). In contrast, AR activity in the *P. viridis* extract in the presence of 0.1 nM DHT ranged from 112 to 340% of the DHT alone, thereby indicating a strong increase in hormone activity in the presence of androgens. Differences in AR activity in the presence of 0.1 nM DHT were significant between sample locations (Kruskal-Wallis, *p* < 0.05). The strongest effects were found in *P. viridis* samples taken from stations M3, M4, and M8 (Figure 1).

ER-α activity in the *P. viridis* extract alone reached 49.6% of 10 nM E_2_ at station M1 and was generally constant in samples from all other locations (18.3% ± 2.2% of 10 nM E_2_). The 10 nM E_2_ estrogenic reference hormone displayed higher ER-α activity in the presence of the *P. viridis* extracts for all locations except M2 and M6, ranging from 98.1 to 216.9% of the activity observed for E_2_ alone. Differences in ER-α activity, in the presence of 1 nM E_2_, were significant between each sample site (Kruskal-Wallis, *p* < 0.05). The greatest increase in ER-α activity was observed for samples taken from stations M3 and M4.

ER-β activity in the *P. viridis* extract alone was more variable than ER-α activity, where peak values were found in samples taken from station M1 (31.3% of 10 nM E_2_) and M4 (16.0% of 10 nM E_2_). The ER-β activity of 10 nM E_2_ in the presence of the *P. viridis* extract ranged from 54.9 to 115.4% of the 10 nM E_2_ alone. ER-β activity in the presence of 1 nM E_2_ in samples M1, M2, M7, and M8 were significantly lower than the activity of E_2_ alone (Kruskal-Wallis, *p* < 0.05) and therefore inhibited the ER-β activity of E_2_. The highest increase in ER-β activity was observed for samples from stations M3, M4, and M6.

### Biological parameters and chemical levels in green mussels.

Biological parameters, levels of specific contaminants in green mussels, and geographical distribution are presented in previous reports (Bayen et al. 2003, 2004). Peaks of POPs and heavy metals were generally found in stations M3, M4, and M8. Biological parameters, such as sex ratio and lipid and moisture content, did not show obvious trends. Ranges are presented in Table 1 for reference.

### Statistical analysis.

The Pearson correlation analysis was used to detect relationships among 23 measured biological and chemical parameters of the *P. viridis* samples. These parameters include sex hormone activity, individual POP and heavy metal contaminant levels, and specimen biological parameters (specimen size, moisture and lipid content, and batch sample sex ratio). In addition, sum concentrations of OCPs (∑OCPs), POPs (∑POPs), and heavy metals (∑HMs) were included in the statistical analysis because these contaminants may exert a combined EDC effect. Heavy metal elements, including Pb, Cd, and Zn, were also measured but are not discussed here because *P. viridis* tissue concentrations were at or below analytical limits of detection. Details on the parameters correlated are given in Table 1. Levels of contaminants are presented as molar concentrations to allow comparison with endocrine activities. The matrix of correlation factors between parameters for Pearson analysis is presented in Table 2.

The Pearson *r* coefficient reveals that ER (both α and β) activities of the *P. viridis* extracts alone have a significantly similar geographical distribution profile (*r* = 0.955, *p* < 0.05). AR and ER-α activities of the green mussel extracts in the presence of the reference hormone also have a similar profile (*r* = 0.928, *p* < 0.05). Statistical analysis reveals that specific individual OCPs, that is, DDTs, chlordanes, and mirex, in *P. viridis* samples have a similar relative concentration profiles among the *P. viridis* tissues from all sample locations (0.895 < *r* < 0.998; *p* < 0.05). On the contrary, OCPs had a different profile than PCBs (*r* = 0.544, *p* > 0.05) and PBDEs (*r* = 0.031, *p* > 0.05).

**Figure 3:**
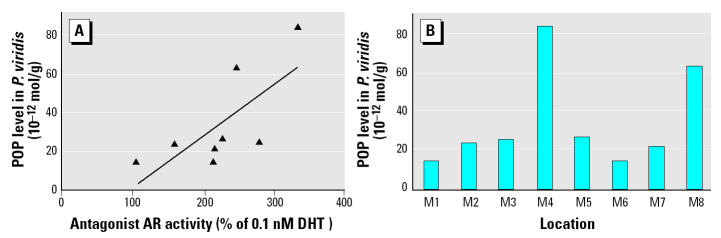
(*A*) Relationship between AR activity in the presence of DHT and total levels of POPs in *P. viridis* tissues (*r* = 0.725; *p* < 0.05). (*B*) Total levels of POPs in green mussel tissues collected around Singapore.

As shown in Figure 2, AR activity in the presence of 0.1 nM DHT is the sex hormone activity with the greatest variability in *P. viridis* tissues between sample locations (i.e., 112–340% of 0.1 nM DHT). AR activity in the presence of 0.1 nM DHT has a significant (*p* < 0.05) and positive correlation with the sum of α- and γ-chlordane levels (*r* = 0.759), as well as the total concentration of POPs (*r* = 0.725; Figure 3). ER-α activity in the presence of 1 nM E_2_ shows similar trends, although the *r* coefficient is weaker and not significant (i.e., *r* = 0.582 with total concentration of POPs). In contrast, activities of the mussel samples in the presence of reference hormones do not show any strong linear correlation with any heavy metal or biological parameters of the mussels (i.e., specimen size, moisture and lipid content, and batch sample sex ratio). Activities of samples alone do not show any strong proportional correlations with either heavy metal or POP tissue concentrations. ER-α and ER-β activities of mussel extracts alone are significantly and negatively correlated with the lipid content of mussel tissues (*r* < −0.749) and positively correlated with moisture content (*r* > 0.728).

## Discussion

### Sex hormone activity distribution in Singapore’s green mussels.

The AR activity of mussel extract alone was very low in samples from all locations (< 1% of 0.1 nM DHT). However, the samples displayed a strong increase in activity in the presence of 0.1 nM DHT, with clear geographical variation, indicating a synergistic response of the mussel extract in the presence of the reference androgenic hormone. The highest increases in AR endocrine activities were found at sample locations close to ship maintenance yards or industrial areas (i.e., stations M4, M3, and M8). The lowest increases in AR activities were found in *P. viridis* samples taken from stations M2 and M6. These sites are adjacent to fish and bivalve aquaculture farms located in the middle of the West and East Straits of Johore and are not directly exposed to industrial and shipping activity.

For ER activity, the mussel extract alone exhibited activities in both ER-α and ER-β bioassays. Endocrine disruption has been previously observed for mussels exposed to environmental pollution, including sewage effluent (Gagné and Blaise 2003). However, it must be noted that E_2_ and other steroids are naturally present in the metabolism of a variety of invertebrates, including oysters and mussels (Matsumoto et al. 1997). Zhu et al. (2003) also detected E_2_ in the gonadal tissues of the blue mussel, *Mytilus edulis*, highlighting its role in the reproductive process of mussels. Therefore, the presence of naturally occurring estrogens in the green mussel may partially account for the variability of activities on ER-α and ER-β receptors found in our study. Finally, the negative significant correlation between ER activities and lipid content might reflect an influence of lipids on the human cell-based bioassay. Therefore, the increase of ER activities for mussel extracts in the presence of E_2_ cannot be clearly interpreted, but it is noteworthy that a similar profile of activity can be observed between sample stations (Figure 2B,D).

Our data suggest that exposure to anthropogenic activities in near-shore coastal waters with reduced hydrodynamic mixing results in a higher EDC load and endocrine activity in *P. viridis*. In a previous study in our laboratory (Gong et al. 2003), sex hormone activities were measured in marine water samples collected from Singapore’s coastal environment. Although differences between exact sample locations and collection time prevent a direct comparison between studies, androgenic and estrogenic peak activities in seawater occurred in confined marine areas and declined rapidly with distance from the coastline. Similarly, investigations of well-known EDCs, including POPs, in harbors in Japan have revealed distinct spatial relationships of contamination, with peak concentrations occurring in the innermost and most confined areas of the harbors (Hosokawa et al. 2003). It is known that coastal waters that receive inputs of pollutants through sewage discharges readily accumulate EDCs in weakly mixed water bodies (Atkinson et al. 2003).

### Relationship between the endocrine profile and POP levels in P. viridis.

In the present study, peaks of AR or ER activity in the presence of the reference hormone corresponded to the sites where heavy metal and POP contamination peak. Pearson correlation analysis shows that the concentration of total POPs (∑POPs) has a positive and significant correlation (*p* < 0.05) with the pattern of the AR activity of the *P. viridis* extracts in the presence of 0.1 nM DHT (Table 2). In contrast, no significant correlation was apparent for sex hormone activities of the mussel extracts in the presence of reference hormones and heavy metal concentrations or any measured biological parameter. This information suggests a relationship between the presence of POPs in the mussel extracts and the androgenic activity of the bioassay (Figure 3A,B). Sex hormone activities in reporter gene assays using human cell lines have been previously assayed for POPs, including chlordanes (Legler et al. 1999), DDT (Legler et al. 1999; Maness et al. 1998), and PCBs (Schrader and Cooke 2003). Endocrine disruption has also been demonstrated for mirex in mice (Dai et al. 2001) and intimated for PBDEs in a study on seals (Hall et al. 2003). Despite concerns over these EDCs, there is no previous report of a bioassay for mirex and PBDEs based on a human cell line. The sum of total POP concentrations in wet mussel tissue ranged from 14 × 10^−12^ to 84 × 10^−12^ mol/g (Table 1). After extraction and dilution, these concentrations correspond to 0.023–0.140 nM used in the bioassay, which are well below threshold concentrations observed for single contaminants previously reported (Legler et al. 1999; Schrader and Cooke 2003). Still, mixtures of single EDCs are known to induce synergistic responses in endocrine bioassays when present at levels below their individual threshold concentrations (Silva et al. 2002).

However, the association between the bioassay and a specific congener should be considered carefully. First, the effects of single chemicals are very complex, and even a single PCB congener, for example, can exhibit both estrogenic and antiestrogenic effects (Gregoraszczuk et al. 2003). Additionally, our extraction technique was designed for monitoring the summation effects of all potential EDCs present in green mussel samples. Other chemicals, including dioxins, alkyl phenols, phthalate esters, toxaphene, contaminant metabolites, estrogenic drugs, and steroids, are all known EDCs (Sonnenschein and Soto 1998) and are likely to be extracted with the solvent mixture (Camel 2000; Schmidt and Steinhart 2002). *In vitro* activity on HeLa cell-based assays are known to be responsive to chemicals such as phthalate esters (Zacharewski et al. 1998) and hydroxylated PCBs (Moore et al. 1997), and many xenobiotic compounds are known to have synergistic endocrine effects (Kortenkamp and Altenburger 1998). The presence of other EDCs in the mussel tissues, such as dioxins, alkyl phenols, or phthalate esters, may therefore account for the remaining variability observed for endocrine activity of the mussel extracts. Therefore, the bioassay should be regarded as a tool to monitor cumulative effects of all potential EDCs in the mussel tissue extracts, which provides a more holistic measure of the impact of complex multichemical mixtures on marine biota.

To our knowledge, this is the first reported use of a human cell-based gene receptor bioassay applied to biological samples for both AR and ER activities in the same sample. Our data show that POP levels and AR activity of the marine mussel extracts, in the presence of 0.1 nM DHT, are significantly and positively related and that the enhanced activity of reference hormones in the presence of biological extracts can be usefully applied as an indicator of EDCs in marine biota.

## Figures and Tables

**Table 1:** *P. viridis* parameters used in Pearson matrix of correlation and range.

Parameter	Range
AR (androgenic activity alone)	0.45–0.85%
ER-α (estrogenic α activity alone)	14.7–49.6%
ER-β (estrogenic β activity alone)	3.4–31.3%
AR + hormone (androgenic activity in presence of hormone)	112–340%
ER-α + hormone (estrogenic α activity in presence of hormone)	98–217%
ER-β + hormone (estrogenic β activity in presence of hormone)	55–116%
As (molar concentration of arsenic)	24–93 × 10^−9^ mol/g
Cr (molar concentration of chromium)	4.2–9.0 × 10^−9^ mol/g
Cu (molar concentration of copper)	53–115 × 10^−9^ mol/g
Ni (molar concentration of nickel)	14–49 × 10^−9^ mol/g
Zn (molar concentration of zinc)	0.39–1.25 × 10^−6^ mol/g
∑HMs (sum of the heavy metal concentrations)	0.49–1.43 × 10^−6^ mol/g
∑CHLs (molar concentration of chlordanes)	1.0–8.1 × 10^−12^ mol/g
∑DDTs (molar concentration of DDTs)	2.2–41.4 × 10^−12^ mol/g
∑PCBs (molar concentration of PCBs)	3.8–44.4 × 10^−12^ mol/g
∑PBDEs (molar concentration of PBDEs)	0.6–16.0 × 10^−12^ mol/g
Mirex (molar concentration of mirex)	0.08–0.62 × 10^−12^ mol/g
∑OCPs (sum of the molar concentrations of mirex, DDTs, and chlordanes)	5.5–50.2 × 10^−12^ mol/g
∑POPs (sum of the molar concentrations of OCPs, PCBs, and PBDEs)	15–84 × 10^−12^ mol/g
Sex ratio (ratio of female to male *P. viridis* samples collected)	0.25–1.00
Size (size of the mussel)	8.4–10.7 cm
Moisture (moisture content of the mussel)	78–86%
Lipid (lipid content of the mussel)	0.7–2.0%

Molar concentrations are based on wet weight.

**Table 2:** Pearson matrix of correlation for 23 measured parameters (biological and chemical) of the *P. viridis* samples.

	ER-α + horm	ER-β+horm	AR + horm	ER-α	ER-β	AR	Cr	Cu	Zn	As	Ni	∑HMs	∑DDTs	∑CHLs	Mirex	∑OCPs	∑PCBs	∑PBDEs	∑POPs	Sex ratio	Size	Moisture	Lipid
Er-α + horm	1	0.530	0.928*	0.094	0.302	0.065	−0.166	0.053	0.087	0.449	−0.248	0.096	0.638	0.650	0.631	0.648	0.145	0.570	0.582	−0.048	−0.145	−0.159	0.278
Er-β + horm		1	0.254	−0.020	0.068	−0.452	−0.149	0.088	−0.206	0.198	−0.154	−0.172	0.354	0.326	0.356	0.355	−0.076	0.397	0.252	−0.651	0.224	0.002	0.091
AR + horm			1	0.030	0.272	0.165	−0.094	0.201	0.319	0.556	−0.214	0.318	0.687	0.759*	0.692	0.707	0.357	0.533	0.725*	0.182	−0.279	−0.252	0.397
ER-α				1	0.955*	0.143	−0.528	−0.668	−0.564	−0.556	−0.318	−0.585	−0.073	−0.314	−0.172	−0.110	−0.238	−0.173	−0.230	−0.058	0.057	0.817*	−0.890*
ER-β					1	0.192	−0.519	−0.500	−0.433	−0.341	−0.372	−0.449	0.213	−0.030	0.113	0.179	−0.044	−0.123	0.057	−0.046	−0.126	0.728*	−0.749*
AR						1	0.300	0.194	0.347	0.164	0.489	0.348	0.269	0.205	0.264	0.264	0.186	−0.450	0.165	0.427	−0.406	0.098	−0.113
Cr							1	0.624	0.503	0.684	0.779*	0.556	0.091	0.279	0.235	0.122	−0.183	0.134	0.000	0.614	−0.075	−0.719*	0.505
Cu								1	0.850*	0.802*	0.543	0.880*	0.527	0.749*	0.630	0.568	0.602	0.032	0.671	0.138	−0.255	−0.583	0.720*
Zn									1	0.640	0.606	0.997*	0.294	0.631	0.401	0.349	0.659	0.153	0.597	0.316	−0.044	−0.482	0.688
As										1	0.348	0.687	0.689	0.860*	0.797*	0.724*	0.283	0.382	0.663	0.407	−0.377	−0.797*	0.785*
Ni											1	0.630	−0.132	0.113	−0.006	−0.097	−0.037	−0.005	−0.079	0.348	0.283	−0.282	0.233
∑HMs												1	0.326	0.657	0.438	0.380	0.632	0.160	0.603	0.327	−0.065	−0.521	0.707*
∑DDTs													1	0.895*	0.985*	0.998*	0.524	−0.010	0.875*	−0.007	−0.708*	−0.221	0.326
∑CHLs														1	0.944*	0.923*	0.616	0.269	0.939*	0.108	−0.453	−0.449	0.615
Mirex															1	0.992*	0.504	0.082	0.880*	0.074	−0.649	−0.346	0.435
∑OCPs																1	0.544	0.031	0.896*	0.010	−0.680	−0.259	0.374
∑PCBs																	1	−0.222	0.820*	−0.194	−0.357	0.022	0.344
∑PBDEs																		1	0.105	0.171	0.479	−0.468	0.465
∑POPs																			1	−0.064	−0.498	−0.239	0.505
Sex ratio																				1	−0.238	−0.476	0.225
Size																					1	0.171	−0.123
Moisture																						1	−0.899*
Lipid																							1

horm, hormone.

*Statistically significant values (*p* < 0.05).
